# Berberine and Oligomeric Proanthocyanidins Exhibit Synergistic Efficacy Through Regulation of PI3K-Akt Signaling Pathway in Colorectal Cancer

**DOI:** 10.3389/fonc.2022.855860

**Published:** 2022-05-04

**Authors:** Keisuke Okuno, Rachana Garg, Yate-Ching Yuan, Masanori Tokunaga, Yusuke Kinugasa, Ajay Goel

**Affiliations:** ^1^ Department of Molecular Diagnostics and Experimental Therapeutics, Beckman Research Institute of City of Hope, Biomedical Research Center, Monrovia, CA, United States; ^2^ Department of Gastrointestinal Surgery, Tokyo Medical and Dental University, Tokyo, Japan; ^3^ Translational Bioinformatics, Center for Informatics, City of Hope, Duarte, CA, United States; ^4^ City of Hope Comprehensive Cancer Center, Duarte, CA, United States

**Keywords:** colorectal cancer, berberine, oligomeric proanthocyanidins, grape seed extract, synergistic effect, apoptosis, MYB, PI3K-Akt signaling pathway

## Abstract

**Background:**

Naturally occurring dietary botanicals offer time-tested safety and anti-cancer efficacy, and a combination of certain compounds has shown to overcome the elusive chemotherapeutic resistance, which is of great significance for improving the mortality of patients with colorectal cancer (CRC). Accordingly, herein, we hypothesized that berberine (BBR) and oligomeric proanthocyanidins (OPCs) might regulate synergistically multiple oncogenic pathways to exert a superior anti-cancer activity in CRC.

**Methods:**

We performed a series of cell culture studies, followed by their interrogation in patient-derived organoids to evaluate the synergistic effect of BBR and OPCs against CRC. In addition, by performing whole genome transcriptomic profiling we identified the key targeted genes and pathways regulated by the combined treatment.

**Results:**

We first demonstrated that OPCs facilitated enhanced cellular uptake of BBR in CRC cells by measuring the fluorescent signal of BBR in cells treated individually or their combination. The synergism between BBR and OPCs were investigated in terms of their anti-tumorigenic effect on cell viability, clonogenicity, migration, and invasion. Furthermore, the combination treatment potentiated the cellular apoptosis in an Annexin V binding assay. Transcriptomic profiling identified oncogene MYB in PI3K-AKT signaling pathway might be critically involved in the anti-tumorigenic properties of the combined treatment. Finally, we successfully validated these findings in patient-derived CRC tumor organoids.

**Conclusions:**

Collectively, we for the first time demonstrate that a combined treatment of BBR and OPCs synergistically promote the anti-tumorigenic properties in CRC possibly through the regulation of cellular apoptosis and oncogene MYB in the PI3K-Akt signaling pathway.

## Introduction

Colorectal cancer (CRC) is one of the most common malignancies worldwide, and currently ranks fourth in disease incidence and the second leading cause of cancer-related deaths, with an estimated 104,270 new cases and 52,980 deaths in 2021 (1). Systemic chemotherapy remains the backbone and one of the best therapeutic options for CRC patients with distant metastasis or recurrence. Although 5-fluorouracil based therapy is the standard of care chemotherapeutic intervention in patients with CRC (2, 3), a large majority of CRC patients tend to acquire resistance and become chemoresistant to such treatments. The acquisition of chemoresistance is primarily due to compensatory overload or emergence of mutations in chemotherapy-induced targeted pathways in a clone of cancer cells (4, 5), which renders these patients unresponsive to subsequent drugs (6, 7). Accordingly, a growing school of thought is that instead of the use of single agents, combination therapies by employing more than one simultaneous drugs that can modulate multiple pathways might enhance the overall therapeutic efficacy as it mitigates the opportunity to develop chemoresistance to a single therapy. In other words, the development of chemoresistance presents a major challenge in the management of CRC, and an optimal combination of various therapeutic modalities is a growing area of research to thwart this clinical challenge.

In recent years, accumulating body of data have shown that *active* principles within various naturally-occurring dietary botanicals offer a time-tested safety and anti-cancer efficacy (8–16). While this list of compounds is quite large, including some of the research undertaken by our research team on curcumin (17–24), Boswellic acids (19, 25, 26), andrographis extract (27–29) and oligomeric proanthocyanidins (OPCs) (27, 30–33). In this regard, OPCs and berberine (BBR), have recently been actively studied for their anti-tumorigenic properties in CRC and other human cancers (16, 27, 30–33). BBR is a small molecule isoquinoline alkaloid extracted from the rhizomes of *Berberis* including Coptidis rhizome and Amur corktree bark and was traditionally used to treat a variety of conditions including bacterial diarrhea in China and native America (16, 34). However, recent studies have shown their diverse pharmacological anti-cancer effects against CRC through a multitude of regulatory mechanisms, including the Wnt signaling pathway, SCAP/SREBP-1 pathway, and β-catenin signaling pathway (35–37). On the other hand, OPCs are a class of polyphenols that are frequently present in a variety of purple or red pigmented plants, including their heavy abundance in the grape seeds. The OPCs also possess anti-cancer properties, and some of the work from our group in the past has demonstrated that their anti-tumorigenic activity in CRC is mediated through cell-cycle dynamics, cancer stem cells, and regulation of ABC drug transporters (30, 32, 33).

A more recent study revealed existence of synergism between BBR and OPCs in patients with type 2 diabetes mellitus (38). This report demonstrated that OPCs inhibited the efflux of BBR and simultaneously increased its cellular uptake by the epithelial cells. Furthermore, OPCs also potentiated the therapeutic efficacy of BBR in mice with diabetes, without causing any harmful adverse effects. On similar lines, our recent research has also demonstrated the cooperative and synergistic effects of OPCs with several other natural compounds, such as curcumin and andrographolide in CRC (27, 31). Furthermore, several previous studies demonstrated the BBR’s synergistic anti-cancer effect with other natural compounds and chemotherapeutic drugs (39–44). In view of these accumulating evidence, we hypothesized that BBR and OPCs might synergistically regulate multiple oncogenic cell signaling pathways to exert a superior anti-cancer activity in CRC. Furthermore, a combination of BBR and OPCs might appropriately also mitigate the likelihood for development of chemoresistance in this malignancy.

Herein, for the first time, we performed a systematic series of experiment in various cell lines, followed by their interrogation in patient-derived organoids to evaluate the synergistic anti-tumorigenic effects of BBR and OPCs in CRC. In addition, we performed whole genome transcriptomic profiling to decipher the molecular mechanisms and key growth regulatory pathways regulated by the combined treatment with BBR and OPCs in this malignancy.

## Materials and Methods

### Cell Culture and Materials

Human colorectal cancer cell lines (RKO and HT29) were purchased from the American Type Culture Collection (Manassas, VA). NCM460 was obtained from INCELL Corporation (San Antonio, TX). All cell lines were tested and authenticated using a panel of genetic and epigenetic markers for their genomic authenticity and tested for mycoplasma on a regular basis. The cell lines were cultured in Dulbecco’s Medium Eagle’s medium (DMEM; Gibco, Carlsbad, CA) containing 10% fetal bovine serum, and 1% penicillin and streptomycin, maintained in a humidified incubator at 37°C in 5% CO_2_, and harvested with 0.05% trypsin-0.03% EDTA (Invitrogen, Carlsbad, CA). Grape seed-OPCs (VX1 extract, EuroPharma USA, Green Bay, WI) and berberine (Berberine HCL, EuroPharma USA, Green Bay, WI) were dissolved in DMSO. Both compounds were diluted to appropriate experimental concentrations in culture medium.

### Cell Viability, Proliferation, Invasion and Wound Healing Assays

Cells were plated at a density of 3-5 x 10^3^ cells per well in 96-well plates under each culture conditions, and incubated with various concentrations of BBR, OPCs, and their combination for 48 hours. We used uniform DMSO concentrations between each treatment groups, including the untreated group. Cell proliferation was measured using Cell Counting Kit-8 (CCK-8) assays (Dojindo, Kumamoto, Japan) as described previously (28). To assess the synergism between BBR and OPCs, the combination index (CI) was calculated using the Chou-Talalay equation (45) at 50% inhibitory concentration. A CI of less than 1.0 was considered to be a synergistic interaction.

For the invasion assay, cells (3-5 x 10^4^ cells) following treatment with BBR, OPCs, and their combination for 48 hours were grown in 24-well transwell chambers (8-μm pore size) coated with Matrigel (BD Biosciences, Franklin Lakes, NJ). After 48 hours, invading cells were detected by Diff-Quik staining. The wound healing assay was performed as described previously (33). Cells were seeded in 12-well plates and grown to 80% confluence. Wounds were created by scraping monolayer cells with a 200-μl pipette tip, and treated with BBR, OPCs, and their combination. 24 hours after scratching, treated and control cells were observed with a microscope.

### Apoptosis and Colony Formation Assays

For the Annexin V binding assay, cells were seeded in 6-well plates, followed by treatment with BBR, OPCs, and their combination for 48 hours. The assay utilizes Annexin V to detect phosphatidylserine on the external membrane of apoptotic cells. After treatment, cells were harvested, and 100μl of cell suspension was added to 100μl of the Muse Annexin V & Dead Cell reagent (Millipore Corp, Billerica, MA). The apoptotic cell fraction was measured using the Muse Cell Analyzer (Millipore Corp) according to the manufacturer’s instructions. The colony formation assay was performed as described previously (25). For this assay, 5 x 10^2^ cells were seeded in 6-well plates, followed by treatment with BBR, OPCs, and their combination for 48 hours. After 7 days, cells were fixed by 100% methanol and stained by 1% crystal violet. The number of colonies with more than 50 cells were counted manually, and the relative change was determined.

### Measurement of BBR Uptake

BBR emits a yellowish fluorescence (excitation: 488nm, emission: 564nm) (46, 47), and its uptake was examined using the Infinite M1000 microplate reader (Tecan Trading AG, Männedorf, Switzerland) and confocal microscopy. The cells were treated with BBR, OPCs, or their combination for 24 hours, and washed two times with cold phosphate buffered solution (PBS) before measurement. BBR autofluorescence was imaged using 488nm excitation and 564nm emission. Cells were observed under 200x magnification using a fluorescent microscope.

### RNA Extraction and Quantitative Reverse Transcription PCR (qRT-PCR)

Total RNA was extracted from cells using the Qiagen miRNeasy Kit (Qiagen, Hilden, Germany), and reverse transcribed to complementary DNA (cDNA) using a high-capacity cDNA Reverse Transcription Kit (Thermo Fischer Scientific, Waltham, MA). The qRT-PCR assays were performed using a SensiFAST SYBR Lo-ROX Kit (Bioline, London, United Kingdom) and the QuantStudio 6/7 Flex RT-PCR System (Applied Biosystems, Foster City, CA). The β-actin gene was used as an internal control. The delta Ct method, where delta Ct is the difference in Ct values between the abundance of target transcripts and the internal control, was used for quantification (48). The primers used in the present study were described in **Supplementary Table S1**.

### Genome-Wide RNA Sequencing and Analysis

Genome-wide RNA sequencing was performed as described previously (30). Total RNA was isolated from RKO and HT29 cells treated with DMSO (Control), BBR, and OPCs for 24 hours using the Qiagen miRNeasy Kit (Qiagen, Hilden, Germany). Next-generation sequencing library construction was performed by the TruSeq RNA library kit (Illumina, Chicago, IL), and the quality of each library was assessed through a High Sensitivity DNA Kit (Agilent, Los Angeles, CA). Libraries were pooled together using a Pippin HT instrument (Sage Science, Beverly, MA). Efficiency of size selection was assessed by a High Sensitivity DNA Kit (Agilent). The pooled libraries were quantified by qRT-PCR using the KAPA Library Quantification Kit, Universal (KAPA Biosystems, Philadelphia, PA) before sequencing on an Illumina HighSeq 2500 with single-end 75 base read lengths. Fastq files were trimmed using Flexbar to remove 3’ bases with quality scores lower than 30 before alignment, and the trimmed reads were mapped to human genome version GRCH38 downloaded from GENCODE57 (49) using HISAT258 (50) to generate alignment files in bam format. Samtools name-sorted bam files (51) were processed using htseq-count to summarize gene level counts (52). DESeq 2 was used for differential gene expression analysis of RNA-sequencing read counts (53).

The raw expression data was analyzed using the Partek Genomic Suite (Build version 10.0.21.0929; Partek Inc., St. Louis, MO). A given gene was considered differentially expressed if it had an FDR step-up *p* < 0.05 and a fold-change > ± 1.5. The heatmap was generated using the Partek Genomic Suite, and the molecular pathways of the differentially expressed genes were analyzed using the KEGG and Partek pathway (Partek, St. Louis, MO) analytical methods. The data was subsequently analyzed for enrichment of GO terms and the KEGG pathways; a pathway was considered significantly enriched if the enrichment score is > 3 and *p* < 0.05.

### Western Blotting

Western blotting (WB) for total protein extracted for cells was performed as described previously (28). Primary antibodies against Bax (1:1000, #5023; Cell Signaling Technology), Bcl-2 (1:1000, #15071; Cell Signaling Technology), Akt (1:1000, #4691; Cell Signaling Technology), and phospho-Akt (Ser473) (1:1000, #4060, Cell Signaling Technology) were used. β-actin (1:1000, #58169; Cell Signaling Technology) were used as an internal control of WB. The relative protein levels were quantified by using the Image-J 1.47v software (http://imagej.nih.gov/ij/index.html).

### Transfection of Small Interfering RNA

For these experiments, 1 x 10^6^ cells were reverse transfected with the MYB siRNA (Thermo Fischer Scientific, Waltham, MA) or negative control siRNA (Thermo Fischer Scientific, Waltham, MA) using Lipofectamine RNAiMAX Transfection Reagent (Thermo Fischer Scientific, Waltham, MA) and then seeded in 6-cm plate. 24-48 hour after transfection, cells were used for the total RNA extraction, total protein extraction, and the other assays.

### Patient-Derived CRC Tumor Organoids

Human primary CRC tissues were obtained from patients with CRC enrolled at Baylor University Medical Center, Dallas, TX. With approval from the ethics committees of the institution, a written informed consent was obtained from all patients. Patients were anonymously coded in accordance with ethical guidelines, as instructed by the Declaration of Helsinki. CRC tumor organoids were cultured using a modified protocol described previously (32). The CRC tumors were incubated in Gentle Cell Dissociation Reagent (STEMCELL Technologies, Vancouver, BC, Canada), and resuspended in DMEM/F-12 with 15mM HEPES (STEMCELL Technologies). Tissues were minced and digested with collagenase solution (5ml of above medium with 75μl collagenase, 124μg/ml dispase type II, and 0.2% Primocen), and then suspended in 100μl of Matrigel (Corning, Tehama County, CA) with 100μl of IntestiCult™ Organoid Growth Medium (#06010, STEMCELL Technologies). The organoids were suspended to four wells such that forms a dome, and then, 750μl of IntestiCult™ Organoid Growth Medium were added to each well. The organoids were randomly assigned into four groups and treated with appropriate concentrations of BBR (20μg/ml), OPCs (20μg/ml), and their combination (BBR: 20μg/ml, OPCs: 20μg/ml). The control organoids were treated with a very low concentration of DMSO. Seven days after growth in the culture medium, the organoids that were about 500 microns in diameter were observed and counted under a bright-field microscope. And then the organoids were harvested by Gentle Cell Dissociation Reagent, and harvested organoids were used for total RNA extractions.

### Statistical Analysis

All experiments were conducted as three independent technical triplicates. All data were expressed as mean ± standard deviation (SD). Two-sided Student’s t test was used to analyze differences between continuous values of each independent group. All *P* values were two-sided, and *P* < 0.05 was considered statistically significant. All statistical analyses were performed using EZR (54), which is a graphical user interface for R (R Foundation for Statistical Computing, Vienna, Austria, version 4.0.3) designed to add statistical functions and is frequently used in biostatistics.

## Results

### OPCs Exhibited a Synergistic Anti-Tumorigenic Effect With BBR in Inhibiting Cell Proliferation, Colony Formation, Migration, and Invasion Through Enhanced Cell Apoptosis

**Figure 1:**
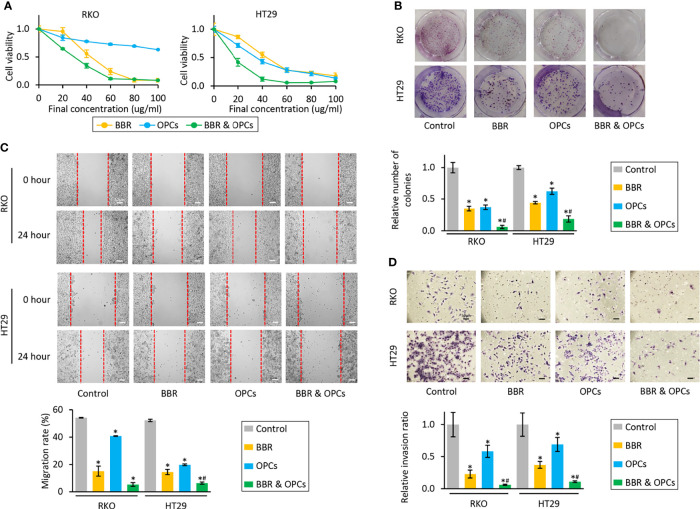
BBR and OPCs exert a synergistic anti-tumorigenic effect in colorectal cancer cell lines. **(A)** CCK-8 assays comparing cell viability following treatment with BBR, OPCs, and their combination for 48 hours in RKO and HT29 cells. Error bars are the mean ± SD. **(B)** Colony formation assays to assess clonogenicity of CRC cells following treatment with BBR, OPCs, and their combination. The average (column) ± SD is indicated (*P < 0.05 vs control, ^#^ *P* < 0.05 vs BBR alone). **(C)** Wound healing assay following treatment with BBR, OPCs, and their combination for 24 hours in RKO and HT29 cells. Photographs show representative scratched and wound recovering areas (marked by red lines). Scale bar = 50μm. The average (column) ± SD is indicated (*P < 0.05 vs control, ^#^ *P* < 0.05 vs BBR alone). **(D)** Invasion assay following treatment with BBR, OPCs, and their combination for 48 hours in RKO and HT29 cells. Scale bar = 50μm. The number of invading cells were counted at four fields randomly selected on the membrane, and then relative invasion ratios were calculated. The average (column) ± SD is indicated (*P < 0.05 vs control, ^#^ *P* < 0.05 vs BBR alone). Photographs show representative fields of invading cells on the membrane (magnification x100).

To evaluate if OPCs exhibit any synergistic anti-tumorigenic effects with BBR in CRC cells, we first investigated the effects of each compound individually (0, 20, 40, 60, 80, and 100μg/ml), and their combination in RKO and HT29 cells. The median IC25 and IC50 of the combination treatment in both cell lines were 11.38 and 23.87μg/ml, respectively, therefore, following experiments were performed at a concentration of 20μg/ml, which is approximately IC50 of a combination treatment. In support of our original hypothesis, the combination of BBR and OPCs demonstrated notably superior anti-proliferative effects in both cells (**Figure 1A**). To assess the synergism between BBR and OPCs, the CI was calculated using the Chou-Talalay equation (45) at 50% inhibitory concentration, with 0.74 and 0.64 value observed in RKO and HT29 cells, respectively (**Supplementary Figure S1**). Because a CI of less than 1.0 was considered to be synergistic, these data indicated a synergistic anti-tumorigenic effect of BBR and OPCs in CRC cells. Interestingly, combination treatment was safe and did not affect cell viability in normal colonic epithelial cell line NCM460 (**Supplementary Figures S2A, B**). Furthermore, the colony formation assays following treatment with BBR (20μg/ml), OPCs (20μg/ml), and their combination (BBR: 20μg/ml, OPCs: 20μg/ml) demonstrated that the combined treatment resulted in a significantly higher reduction of clonogenicity than either treatment alone in CRC cells (fold change [FC] = 0.22 vs BBR alone, *P* = 0.01; FC = 0.21 vs OPCs alone, *P* = 0.01 in RKO cells; FC = 0.51 vs BBR alone, *P* = 0.04; FC = 0.35 vs OPCs alone, *P* = 0.04 in HT29 cells; **Figure 1B**).

Next, we assessed the ability of BBR and OPCs to inhibit cellular migration and invasion, which is critical for cancer progression and metastases. With regards to the wound healing and invasion assay, BBR and OPCs significantly decreased cell migration and invasion compared to control cells, while the combination treatment of these compounds significantly enhanced these effects for their ability to inhibit cell migration (in RKO cells; vs BBR alone, *P* = 0.15; vs OPCs alone, *P* = 0.01; In HT-29 cells, vs BBR alone, *P* = 0.05; vs OPCs alone, *P* < 0.01; **Figure 1C**) and invasion (vs BBR alone, *P* = 0.03; vs OPCs alone, *P* < 0.01 in RKO cells; vs BBR alone, *P* < 0.01; vs OPCs alone, *P* < 0.01 in HT29 cells; **Figure 1D**).

**Figure 2:**
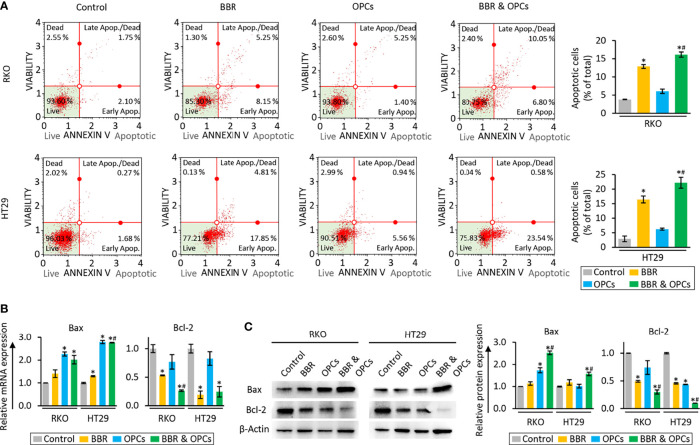
BBR and OPCs promoted the cell apoptosis in colorectal cancer cell lines. **(A)** Representative images of cells undergoing apoptosis that stained for annexin V assay in RKO and HT29 cells. The average (column) ± SD is indicated (*P < 0.05 vs control, ^#^ *P* < 0.05 vs BBR alone). **(B)** qRT-PCR analysis of Bax and Bcl-2 expression in RKO and HT29 cells following treatment with BBR, OPCs, and their combination for 48 hours. Relative expression was calculated using β-Actin mRNA expression as an internal control. The average (column) ± SD is indicated (*P < 0.05 vs control, ^#^ *P* < 0.05 vs BBR alone). **(C)** WB of Bax and Bcl-2 expression in RKO and HT29 cells following treatment with BBR, OPCs, and their combination for 48 hours. β-Actin protein was used as an internal control. The average (column) ± SD is indicated (*P < 0.05 vs control, ^#^ *P* < 0.05 vs BBR alone).

To clarify the underlying mechanism that orchestrated the enhanced efficacy for the combination treatment of BBR and OPCs in reducing the cell viability and clonogenicity, we evaluated their combinatorial impact on apoptotic rates by an Annexin V binding assay. Compared to the untreated group, BBR significantly increased the rates of apoptosis, whereas the combined treatment with BBR and OPCs further enhanced the apoptotic potential of BBR (**Figure 2A**). Furthermore, for the expression of apoptosis related genes, the combination treatment significantly up-regulated Bax and down-regulated Bcl-2 at the mRNA and protein level (**Figures 2B, C**). Taken together, these data confirm that OPCs potentiated the anti-tumorigenic effect of BBR through increased apoptosis and other key cancer cell death mechanism in CRC cells.

### OPCs Increased the Cellular Uptake of BBR in CRC Cells

**Figure 3:**
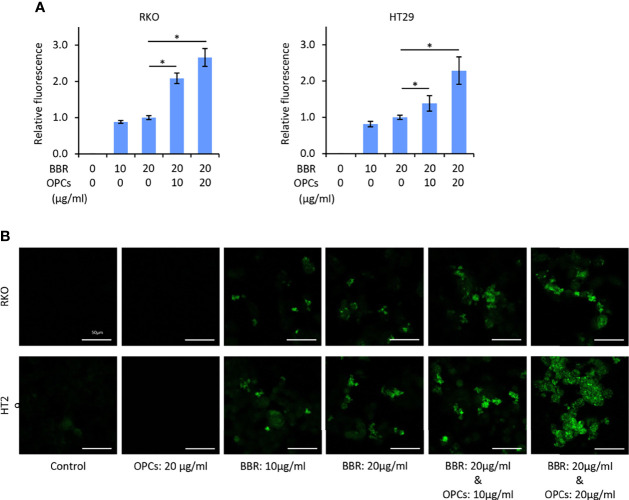
OPCs increased the cellular uptake of BBR in colorectal cancer cell lines. **(A)** Cellular fluorescence intensity value of BBR following treatment with BBR and combination of BBR and OPCs for 24 hours in RKO and HT29 cells. The average (column) ± SD is indicated (*P < 0.05). **(B)** Representative images of RKO and HT29 cells following treatment with BBR, OPCs, and their combination for 24 hours using a fluorescent microscope under 200x magnification with a 488 nm/564 nm excitation filter (scale bar = 50μm).

To evaluate the mechanism of synergism, we examined whether OPCs affect the cellular uptake of BBR in CRC cells. Because the IC25 and IC50 concentration of the combination treatment was approximately 10 and 20μg/ml, respectively (**Figure 1A**), RKO and HT29 cell lines were treated with BBR (10 and 20μg/ml) and various concentration of OPCs (10 and 20μg/ml) for 24 hours. Using BBR’s yellowish fluorescence (excitation: 488nm, emission: 564nm), the cellular uptake of BBR was visualized by the multimode microplate reader and confocal microscopy. Interestingly, we noted that OPCs promoted the fluorescence intensity of BBR in both RKO and HT29 cells, while the combination of OPCs exhibited significantly higher uptake of BBR than BBR alone in a concentration-dependent manner; the combination of 20μg/ml OPCs revealed 2.91 and 2.28 times higher uptake of BBR in RKO and HT29 cells, respectively (*P* = 0.04 and *P* = 0.03, respectively, vs BBR: 20μg/ml alone; **Figures 3A, B**). These data suggest that OPCs facilitate increased cellular uptake of BBR in CRC cells, supporting the previous observation made in type 2 diabetes mellitus (38).

### Transcriptomic Profiling Revealed That BBR and OPCs Modulated Key Cancer-Associated Pathways

**Figure 4:**
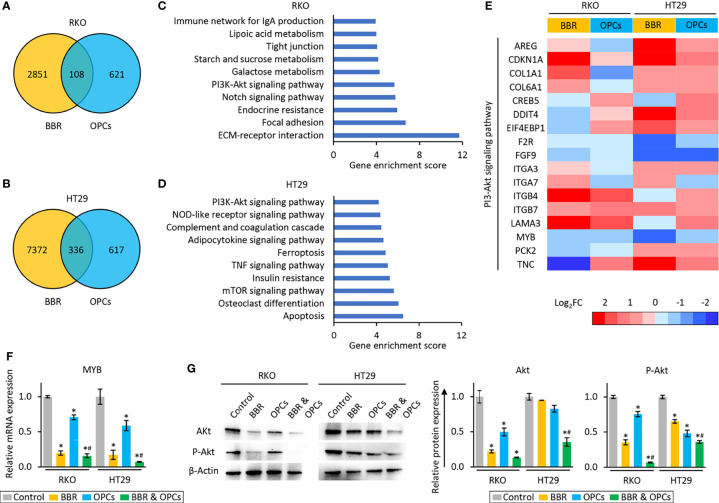
BBR and OPCs modulated multiple cancer-associated pathways. **(A, B)** Venn-diagram of dysregulated expression (> 1.5-fold) of the genes following treatment with BBR and OPCs in RKO **(A)** and HT29 **(B)** cells. **(C, D)** The top 10 pathways affected by both BBR and OPCs in RKO **(C)** and HT29 **(D)** cells. **(E)** Heatmap of differentially expressed genes regulated by both BBR and OPCs in PI3K-Akt signaling pathway. **(F)** qRT-PCR analysis of MYB expression in RKO and HT29 cells following treatment with BBR, OPCs, and their combination for 48 hours. Relative expression was calculated using β-Actin mRNA expression as an internal control. The average (column) ± SD is indicated (*P < 0.05 vs control, ^#^ *P* < 0.05 vs BBR alone). **(G)** WB of Akt and phospho-Akt (ser473) (p-Akt) expression in RKO and HT29 cells following treatment with BBR, OPCs, and their combination for 48 hours. β-Actin protein was used as an internal control. The average (column) ± SD is indicated (*P < 0.05 vs control, #P < 0.05 vs BBR alone).

To unravel the molecular mechanisms underlying the anti-tumorigenic properties of the combination treatment, we examined the genome-wide transcriptomic changes induced by BBR and OPCs in RKO and HT29 cells using the Partek Genomic Suite. The treatment of BBR resulted in 2851 up- or down-regulated (> 1.5-fold) probes in RKO cells, and 7372 up- or down-regulated (> 1.5-fold) probes in HT29 cells. We also detected that 621 and 617 up- or down-regulated (> 1.5-fold) probes by the treatment of OPCs in RKO and HT29, respectively. Among them, 108 and 336 genes were altered by both treatments in RKO and HT29, respectively (**Figures 4A, B**).

Next, we examined Gene Ontology enrichment analysis of these up- or down-regulated genes using the KEGG and Partek pathway analytical methods. This analysis revealed that PI3K-Akt signaling pathway was one of the key pathways that was commonly abrogated in both RKO and HT29 cells (enrichment score: 5.67, *p* < 0.01 in RKO; enrichment score: 4.20, *p* = 0.01 in HT29), because PI3K-Akt signaling pathway was the only pathway that was common among the top 10 pathways in both cells (**Figures 4C, D**). Since the expression of CDKN1A, F2R, FGF9, ITGB7, and MYB were up- or down-regulated by both treatments in each cell line (**Figure 4E**), we next validated the expression of these 5 genes by qRT-PCR, and the results were consistent with the sequencing data (**Figure 4F** and **Supplementary Figure S3**). Furthermore, the protein expression level of Akt, which is one of the key genes in the PI3K-Akt signaling pathway, was significantly inhibited by combined treatment of BBR and OPCs in both cells. In addition, combined treatment attenuated phosphorylation of Akt at Ser473, which indicated that the PI3K-Akt signaling pathway is the key pathway regulated by combined treatment of BBR and OPCs (**Figure 4G**). For the combination treatment, the expression of only MYB were significantly down-regulated compared to control and BBR in both cells. MYB emerged as one of the key regulators of this anti-cancer activity. MYB, encodes three types of oncoprotein that function as a transcription regulator, is considered to be an oncogene, and is involved in the regulation of apoptotic process (55–58) – hence, supporting the apoptotic potential of the combination treatment (**Figures 2A–C**). Furthermore, MYB is often up-regulated in patients with CRC (59, 60), and recently have gained increased attention as a potential and novel therapeutic target in cancer (61, 62). Based upon these findings, among these five genes, we focused on MYB as a target of the combined treatment with BBR and OPCs and selected this gene for subsequent experiments.

### SiRNA-Mediated Knockdown of MYB Exhibited the Anti-Tumorigenic Effects by Inhibiting Cell Proliferation and Migration in CRC Cells

**Figure 5:**
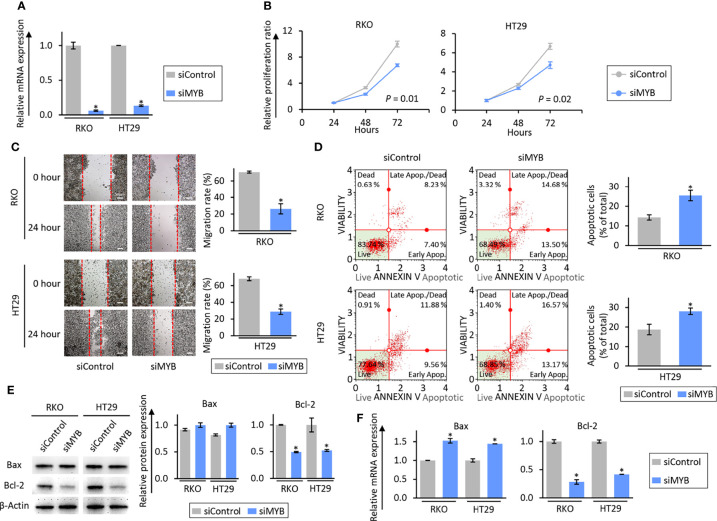
The siRNA-based knock-down of MYB inhibited a tumorigenic effect through induction of apoptosis in colorectal cancer cell lines. **(A)** qRT-PCR analysis of MYB expression in RKO and HT29 cells. MYB expression was knock-downed by siMYB. Relative expression was calculated using β-Actin mRNA expression as an internal control. The average (column) ± SD is indicated (*P < 0.05). **(B)** Cell proliferation assay in RKO and HT29 cells after MYB knock-down. Total viable cells were measured with a CCK-8 assay on the indicated days. Error bars are the mean ± SD. **(C)** Wound healing assay in RKO and HT29 cells after MYB knock-down. Photographs show representative scratched and wound recovering areas (marked by red lines). Scale bar = 50μm. The average (column) ± SD is indicated (*P < 0.05). **(D)** Representative images of cells undergoing apoptosis that stained for annexin V assay in RKO and HT29 cells after MYB knock-down. The average (column) ± SD is indicated (*P < 0.05). **(E)** WB of Bax and Bcl-2 expression in RKO and HT29 cells after MYB knock-down. β-Actin protein was used as an internal control. The average (column) ± SD is indicated (*P < 0.05). **(F)** qRT-PCR analysis of Bax and Bcl-2 expression in RKO and HT29 cells after MYB knock-down. Relative expression was calculated using β-Actin mRNA expression as an internal control. The average (column) ± SD is indicated (*P < 0.05).

To address the role of MYB in the anti-tumorigenic properties of the combination treatment in CRC cells, we next performed siRNA-based knockdown of MYB in RKO and HT29 cells. The expression of MYB at the mRNA levels was inhibited 24 hours after transfection of its siRNA (**Figure 5A**). As for series of proliferation and wound healing assays, knockdown of MYB significantly inhibited cell proliferation (*P* = 0.01 in RKO cells; *P* = 0.02 in HT29 cells; **Figure 5B**) and migration (FC = 0.37, *P* = 0.05 in RKO cells; FC = 0.42, *P* = 0.04 in HT29 cells; **Figure 5C**) in RKO and HT29 cells. We also evaluated its impact on apoptotic rates *via* an Annexin V binding assay to further clarify that knockdown of MYB reduced the cell viability and migration. Compared to CRC cells transfected negative control siRNA, knockdown of MYB significantly increased the rates of cellular apoptosis (FC = 1.78, *P* = 0.04 in RKO cells; FC = 1.50, *P* = 0.03 in HT29 cells; **Figure 5D**). Moreover, knockdown of MYB significantly up-regulated Bax expression and down-regulated Bcl-2 expression at the mRNA and protein levels (**Figures 5E, F**). Taken together, these data suggest that MYB plays an important role in cancer progression and metastases through regulation of cellular apoptosis, and this gene is critically involved in the anti-tumorigenic effects of the combined treatment with BBR and OPCs.

### The Combination of BBR and OPCs Synergistically Inhibited the Growth in Patient-Derived Organoids

**Figure 6:**
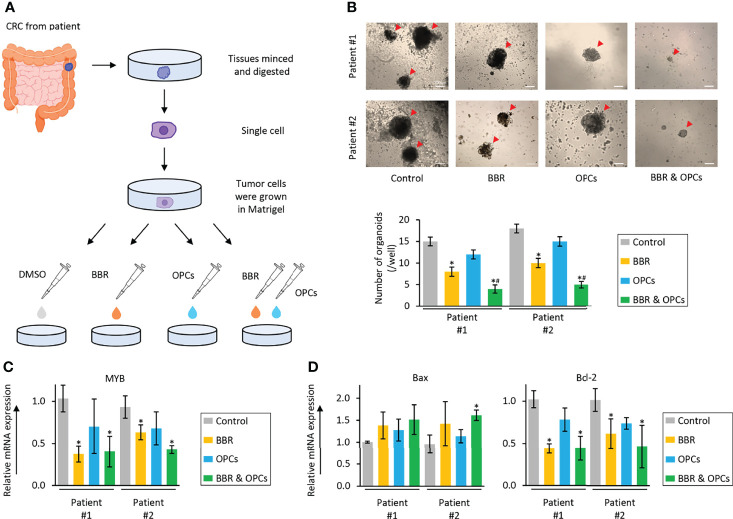
The combination of BBR and OPCs effectively suppressed growth of tumor organoids derived from human colorectal cancers. **(A)** Schematic protocol of BBR, OPCs, and their combination treatment on tumor organoids derived from human colorectal cancers. **(B)** Representative images of tumor organoids following treatment with BBR, OPCs, and their combination. Scale bar = 500μm. The average (column) ± SD is indicated (*P < 0.05 vs control, ^#^ *P* < 0.05 vs BBR alone). **(C)** qRT-PCR analysis of MYB expression in tumor organoids following treatment with BBR, OPCs, and their combination. Relative expression was calculated using β-Actin mRNA expression as an internal control. The average (column) ± SD is indicated (*P < 0.05 vs control). **(D)** qRT-PCR analysis of Bax and Bcl-2 expression in tumor organoids following treatment with BBR, OPCs, and their combination. Relative expression was calculated using β-Actin mRNA expression as an internal control. The average (column) ± SD is indicated (*P < 0.05 vs control).

Tumor organoid model and 3D primary cultures are physiologically superior to the conventional monolayer cultured cells for the study of anti-cancer agents (63). Thus, in order to further support our cell culture findings, we finally utilized a tumor-derived organoid model from patients with CRC. The tumor organoids were treated with BBR (20μg/ml), OPCs (20μg/ml), and their combination (BBR: 20μg/ml; OPCs: 20μg/ml) for 1 week (**Figure 6A**). It was quite reassuring to witness that the results in this experimental model were consistent with those seen in cell culture experiments, where BBR significantly inhibited the growth and formation of patient-derived organoids compared to untreated ones. Furthermore, the combined treatment with BBR and OPCs enhanced the anti-tumorigenic potentials in patient-derived organoids (FC = 0.50, *P* = 0.04 in patient #1; FC = 0.50, *P* = 0.03 in patient #2; **Figure 6B**). In support of our phenotypic observations made in tumor-derived organoids, the expression of MYB was also significantly down-regulated by the combined treatment of BBR and OPCs (FC = 0.40 vs control, *P* = 0.05 in patient #1; FC = 0.43 vs control, *P* = 0.03 in patient #2; **Figure 6C**). Interestingly, for the expression of apoptosis related genes, Bax was also significantly up-regulated, and Bcl-2 was down-regulated by the combination treatment (**Figure 6D**). Collectively, these data highlight that the combination treatment with BBR and OPCs possess a remarkable anti-tumorigenic effect in inhibiting organoid growth by enhancing cellular apoptosis and by downregulating the expression of MYB *via* the PI3K-Akt signaling pathway.

## Discussion

In treating patients with CRC, overcoming the intrinsic and acquired drug resistance is one of the most challenging problems because chemoresistance leads to frequent cancer recurrence, its dissemination to other organs, and patient death. Furthermore, there is an unequivocal consensus that chemoresistant mutants generally exist as clones at very low numbers before the initiation of chemotherapy. However, following initiation of a single-drug treatment, it often leads to the selection and evolution of such drug-resistant mutational populations of cancer cells, and subsequent treatment with other drugs is usually ineffective due to the establishment of such resistant mechanisms (6, 7). In contrast, combined treatment with multiple drugs presumably impacts multiple pathways, yields a better therapeutic efficacy, which is often attributed to the smaller chances for the emergence of chemoresistance to multiple drugs in cancer cells. However, the therapeutic benefits of combined treatment are usually accompanied by the simultaneous drug toxicity, as well as increased costs – which often limits their clinical efficacy.

**Figure 7:**
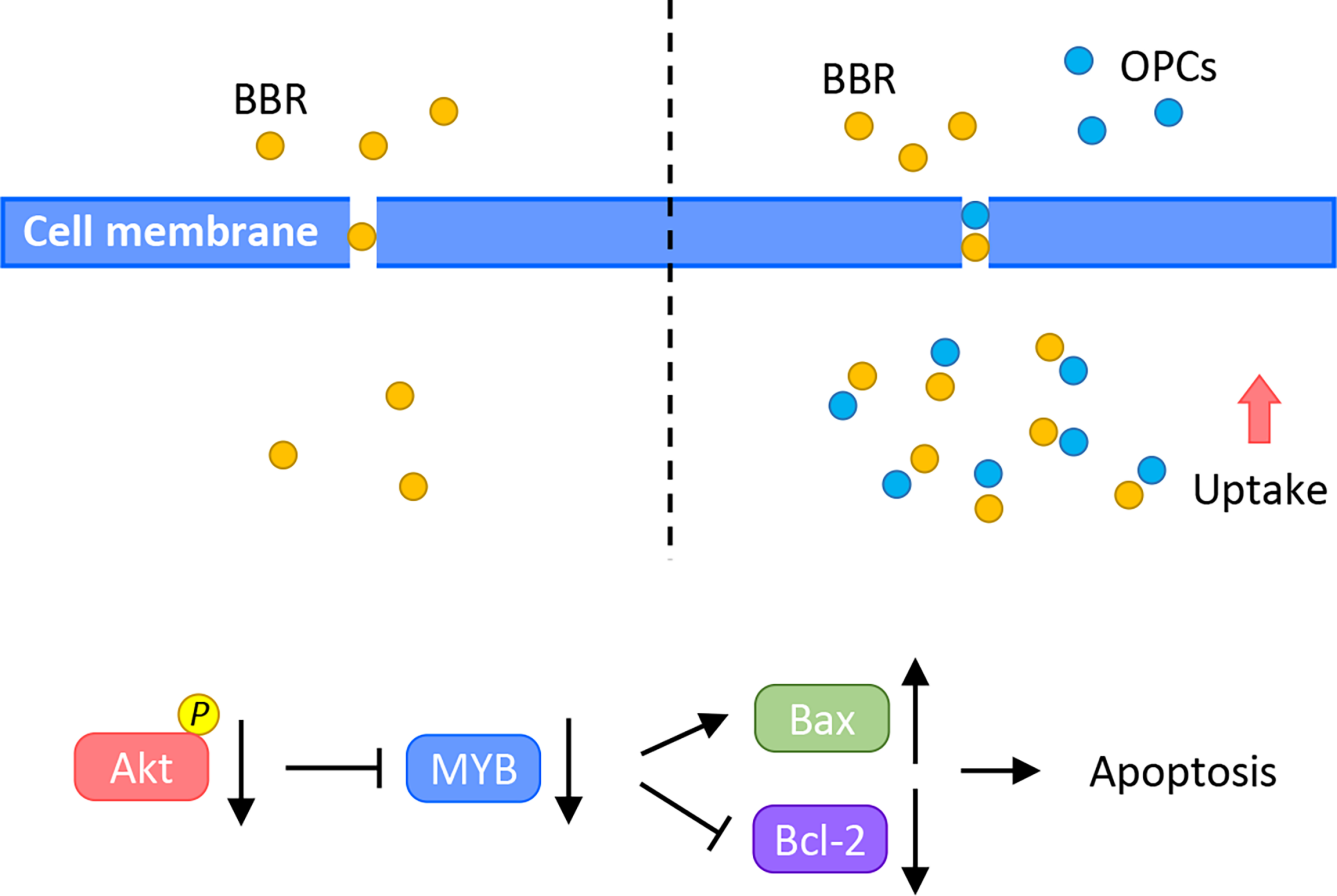
A schematic illustration of the synergistic anti-cancer effect of BBR and OPCs. This illustration demonstrates treatment of BBR alone (left) and combined treatment of BBR and OPCs (right) in CRC cells. OPCs increased the cellular uptake of BBR in CRC cells and enhanced BBR’s anti-cancer potential through MYB and PI3K-Akt signaling pathway.

To overcome these clinical challenges, the use of safe and cost-effective diet-based natural botanicals offer a time-tested safety and anti-cancer efficacy. Actually, 75 of 175 small molecules recognized as anti-cancer drugs currently, can be traced back to their origin to naturally-occurring botanicals, from 1981 to 2019 (64). Furthermore, several natural compounds have recently gained increasing attention due to their anti-cancer potential through their ability to target multiple oncogenic pathways, in a safe manner (13–16, 65, 66). Among this list of many such compounds including curcumin, boswellia, resveratrol and andrographis, BBR and OPCs have recently been interrogated due to their promising anti-tumorigenic properties in CRC (30, 32, 33, 35–37). In fact, a recent study also reported a synergistic effect between these two natural compounds in type 2 diabetes mellitus (38). Accordingly, in this study, we for the first time investigated the synergistic anti-tumorigenic effect of BBR and OPCs in CRC by enhancing cellular apoptosis and by downregulating the expression of MYB *via* the PI3K-Akt signaling pathway (**Figure 7**), using a series of systematic cell culture and patient-derived tumor organoid experimental models. Furthermore, considering that our observed effects for BBR and OPCs were comparable in microsatellite unstable (MSI) and microsatellite stable (MSS) cell lines, this suggests that these compounds can be used for almost all patients with colorectal cancer; hence highlighting their broader application for this malignancy.

Current evidence has demonstrated the cooperative anti-tumorigenic effects of OPCs with other natural compounds and various chemotherapeutic drugs. Our previous studies also reported that by combining OPCs with curcumin and andrographis, enhanced their growth inhibitory activity in CRC cells (27, 31). In addition, we previously demonstrated the additional ability of OPCs to overcome chemoresistance through the suppression of ABC transporters in CRC (30). In the current study, we build upon our previous work, and illustrate that OPCs also demonstrated synergism with BBR in CRC cells, and in part, this efficacy was perhaps driven by the ability of OPCs to increase the cellular uptake of BBR in cancer cells. Procyanidins including OPCs are considered to downregulate the expression of P-glycoprotein, which is an important protein of the cell membrane that pumps many foreign substances out of cells, by inhibiting NF-κB activation and MAPK/ERK-mediated YB-1 activity (38, 67). These previous studies and our present results suggest that the synergism observed between BBR and OPCs might be a result of OPCs to facilitate the increased cellular uptake of BBR by inhibiting the efflux of BBR through the regulation of P- glycoprotein.

The PI3K-Akt signaling pathway plays a vital role in cancer development and drug-resistance, and BBR’s properties on the cell apoptosis through the regulation of this signaling pathway have been illustrated in various types of cancers (68, 69). In osteosarcoma and breast cancer, BBR promoted cellular apoptosis through inhibition of the PI3K-Akt signaling pathway (70, 71). More interestingly, BBR acted synergistically with PI3K inhibitor in SW480 CRC cells (72). In line with previous reports, in our present study, BBR enhanced cellular apoptosis in CRC cells and regulated the PI3K-Akt signaling pathway. Furthermore, the combination of OPCs further enhanced the anti-tumorigenic apoptotic potential of BBR. Within the context of this pathway, MYB, encodes for three types of oncoproteins and functions, is a transcriptional regulator in the PI3K-Akt signaling pathway and considered to be an oncogene that regulates apoptosis (55–58). Accordingly, in this study, we focused on the evaluation of the function of MYB as a potential target in support of the combined treatment with BBR and OPCs. According, in support of several previous reports and our results of siRNA experiments in this study, MYB played an important role in cancer progression and metastases through regulation of apoptosis. Although transcriptional factors are traditionally considered difficult to target, renewed interest in targeting transcriptional factors has opened a new horizon in the field of cancer drug discovery. More specifically, MYB has also emerged as an attractive therapeutic target in cancers, and several small molecules or peptide-mimetic inhibitors have shown promise as anti-cancer agents (61, 62). However, none of these compounds have thus far been used in the clinical practice due to the accompanying toxicity. In this study, we for the first time report that MYB is critically involved in the anti-tumorigenic effects for the observed anti-cancer effects with BBR and OPCs. Our new findings thus offer a potentially safe alternative to the drug toxicity of MYB-inhibitors, considering the time-tested safety and recently reported anti-cancer efficacy of these nutraceuticals.

In this study, for the first time, we investigated the synergistic anti-tumorigenic effect of BBR and OPCs against CRC cells, however, we would like to acknowledge some of the potential limitations of our work. Since the primary objective of our study was to investigate the synergism of BBR and OPCs against CRC cells, we did not perform detailed molecular functional studies. In addition, given the constraints with tumor-derived organoids, we were able to analyze such organoids from only two patients. Moreover, our pathway analyses were performed in cells treated by BBR alone and OPCs alone, not the combined treatment. While RNA sequencing of the combined treatment might reveal additional genes and pathways that orchestrate the chemopreventive effects of these compounds, the primary objective of the present study was the gain first insights on the synergistic anti-cancer effect of BBR and OPCs against CRC cells. Therefore, we first performed the RNA sequencing in cells treated by BBR alone and OPCs alone. Future studies with such combinatorial treatments and transcriptomic profiling might reveal previously unrecognized genes and regulatory pathways in colorectal cancer. Although, we validated our *in vivo* findings about the synergism of BBR and OPCs in patient-derived tumor organoids experiments, this study involved no animal studies to demonstrate the toxicity examination. Accordingly, future studies are warranted to further investigate the detailed molecular mechanisms of the combined treatment with BBR and OPCs on MYB and PI3K-Akt signaling pathway, including the validation of our findings in the tumor spheroids and animal models.

## Conclusions

In conclusion, we have firstly demonstrated that the combination treatment of BBR and OPCs promoted the anti-tumorigenic effects in CRC possibly through the inhibition of cellular apoptosis by regulating the expression of MYB in the PI3K-Akt signaling pathway. Our findings could provide essential evidence of the combined treatment with BBR and OPCs as a potential therapeutic option for patients with CRC.

## Data Availability Statement

The original contributions presented in the study are included in the article/**Supplementary Material**. Further inquiries can be directed to the corresponding author.

## Author Contributions

KO, RG, MT, YK, and AG conceived and designed the experiments. KO and RG performed the experiments and analyzed the data. KO and AG wrote the manuscript. All authors contributed to the article and approved the submitted version.

## Funding

This work was supported by, CA184792, CA187956, CA227602, CA072851, and CA202797 grants from the National Cancer Institute, National Institutes of Health.

## Conflict of Interest

The authors declare that the research was conducted in the absence of any commercial or financial relationships that could be construed as a potential conflict of interest.

## Publisher’s Note

All claims expressed in this article are solely those of the authors and do not necessarily represent those of their affiliated organizations, or those of the publisher, the editors and the reviewers. Any product that may be evaluated in this article, or claim that may be made by its manufacturer, is not guaranteed or endorsed by the publisher.

## Supplementary Material

Supplementary Figure 1 Isobologram analysis after combined treatment with BBR and OPCs in RKO and HT29 cells.

Supplementary Figure 2
**(A)** CCK-8 assays comparing cell viability following treatment with BBR, OPCs, and their combination for 48 hours in NCM460 cells. Error bars are the mean ± SD. **(B)** Cell viability following treatment with BBR (20μg/ml), OPCs (20μg/ml), and their combination (BBR: 20μg/ml and OPCs: 20μg/ml) for 48 hours in NCM460 cells. The average (column) ± SD is indicated.

Supplementary Figure 3 The qRT-PCR analysis of CDKN1A, F2R, FGF9, and ITGB7 expression in RKO and HT29 cells following treatment with BBR, OPCs, and their combination for 48 hours. Relative expression was calculated using β-Actin mRNA expression as an internal control. The average (column) ± SD is indicated (*P < 0.05, #P < 0.05 vs BBR alone).

## References

[1] Siegel RL Miller KD Fuchs HE Jemal A. Cancer Statistics, 2021. CA Cancer J Clin (2021) 71(1):7–33. doi: 10.3322/caac.21654 33433946

[2] Giacchetti S Perpoint B Zidani R Le Bail N Faggiuolo R Focan C. Phase III Multicenter Randomized Trial of Oxaliplatin Added to Chronomodulated Fluorouracil-Leucovorin as First-Line Treatment of Metastatic Colorectal Cancer. J Clin Oncol (2000) 18(1):136–47. doi: 10.1200/JCO.2000.18.1.136 10623704

[3] Colucci G Gebbia V Paoletti G Giuliani F Caruso M Gebbia N. Phase III Randomized Trial of FOLFIRI Versus FOLFOX4 in the Treatment of Advanced Colorectal Cancer: A Multicenter Study of the Gruppo Oncologico Dell'Italia Meridionale. J Clin Oncol (2005) 23(22):4866–75. doi: 10.1200/JCO.2005.07.113 15939922

[4] Bernards R. A Missing Link in Genotype-Directed Cancer Therapy. Cell (2012) 151(3):465–8. doi: 10.1016/j.cell.2012.10.014 23101617

[5] Yamaguchi H Chang SS Hsu JL Hung MC. Signaling Cross-Talk in the Resistance to HER Family Receptor Targeted Therapy. Oncogene (2014) 33(9):1073–81. doi: 10.1038/onc.2013.74 PMC3874419 23542173

[6] Komarova NL Boland CR. Cancer: Calculated Treatment. Nature (2013) 499(7458):291–2. doi: 10.1038/499291a PMC3831845 23868257

[7] Diaz LA Jr Williams RT Wu J Kinde I Hecht JR Berlin J. The Molecular Evolution of Acquired Resistance to Targeted EGFR Blockade in Colorectal Cancers. Nature (2012) 486(7404):537–40. doi: 10.1038/nature11219 PMC3436069 22722843

[8] Bishayee A Sethi G. Bioactive Natural Products in Cancer Prevention and Therapy: Progress and Promise. Semin Cancer Biol (2016) 40-41:1–3. doi: 10.1016/j.semcancer.2016.08.006 27565447

[9] Ranjan A Ramachandran S Gupta N Kaushik I Wright S Srivastava S. Role of Phytochemicals in Cancer Prevention. Int J Mol Sci (2019) 20(20):4981. doi: 10.3390/ijms20204981 PMC6834187 31600949

[10] Choudhari AS Mandave PC Deshpande M Ranjekar P Prakash O. Phytochemicals in Cancer Treatment: From Preclinical Studies to Clinical Practice. Front Pharmacol (2019) 10:1614. doi: 10.3389/fphar.2019.01614 32116665 PMC7025531

[11] Cháirez-Ramírez MH de la Cruz-López KG García-Carrancá A. Polyphenols as Antitumor Agents Targeting Key Players in Cancer-Driving Signaling Pathways. Front Pharmacol (2021) 12:710304. doi: 10.3389/fphar.2021.710304 34744708 PMC8565650

[12] Haque A Brazeau D Amin AR. Perspectives on Natural Compounds in Chemoprevention and Treatment of Cancer: An Update With New Promising Compounds. Eur J Cancer (2021) 149:165–83. doi: 10.1016/j.ejca.2021.03.009 PMC8113151 33865202

[13] Ratajczak K Borska S. Cytotoxic and Proapoptotic Effects of Resveratrol in In Vitro Studies on Selected Types of Gastrointestinal Cancers. Molecules (2021) 26(14):4350. doi: 10.3390/molecules26144350 34299624 PMC8305210

[14] Sampaio LA Pina LTS Serafini MR Tavares DDS Guimarãães AG. Antitumor Effects of Carvacrol and Thymol: A Systematic Review. Front Pharmacol (2021) 12:702487. doi: 10.3389/fphar.2021.702487 34305611 PMC8293693

[15] Naji M Soroudi S Akaberi M Sahebkar A Emami SA. Updated Review on the Role of Curcumin in Gastrointestinal Cancers. Adv Exp Med Biol (2021) 1308:55–89. doi: 10.1007/978-3-030-64872-5_6 33861437

[16] Wang Y Liu Y Du X Ma H Yao J. The Anti-Cancer Mechanisms of Berberine: A Review. Cancer Manag Res (2020) 12:695–702. doi: 10.2147/CMAR.S242329 32099466 PMC6996556

[17] Toden S Goel A. The Holy Grail of Curcumin and its Efficacy in Various Diseases: Is Bioavailability Truly a Big Concern? J Restor Med (2017) 6(1):27–36. doi: 10.14200/jrm.2017.6.0101 30899605 PMC6424351

[18] Toden S Theiss AL Wang X Goel A. Essential Turmeric Oils Enhance Anti-Inflammatory Efficacy of Curcumin in Dextran Sulfate Sodium-Induced Colitis. Sci Rep (2017) 7(1):814. doi: 10.1038/s41598-017-00812-6 28400554 PMC5429743

[19] Toden S Okugawa Y Buhrmann C Nattamai D Anguiano E Baldwin N. Novel Evidence for Curcumin and Boswellic Acid-Induced Chemoprevention Through Regulation of miR-34a and miR-27a in Colorectal Cancer. Cancer Prev Res (Phila) (2015) 8(5):431–43. doi: 10.1158/1940-6207.CAPR-14-0354 PMC4417447 25712055

[20] Link A Balaguer F Shen Y Lozano JJ Leung HC Boland CR. Curcumin Modulates DNA Methylation in Colorectal Cancer Cells. PloS One (2013) 8(2):e57709. doi: 10.1371/journal.pone.0057709 23460897 PMC3584082

[21] Goel A Aggarwal BB. Curcumin, the Golden Spice From Indian Saffron, is a Chemosensitizer and Radiosensitizer for Tumors and Chemoprotector and Radioprotector for Normal Organs. Nutr Cancer (2010) 62(7):919–30. doi: 10.1080/01635581.2010.509835 20924967

[22] Goel A Jhurani S Aggarwal BB. Multi-Targeted Therapy by Curcumin: How Spicy is it? Mol Nutr Food Res (2008) 52(9):1010–30. doi: 10.1002/mnfr.200700354 18384098

[23] Goel A Kunnumakkara AB Aggarwal BB. Curcumin as "Curecumin": From Kitchen to Clinic. Biochem Pharmacol (2008) 75(4):787–809. doi: 10.1016/j.bcp.2007.08.016 17900536

[24] Goel A Boland CR Chauhan DP. Specific Inhibition of Cyclooxygenase-2 (COX-2) Expression by Dietary Curcumin in HT-29 Human Colon Cancer Cells. Cancer Lett (2001) 172(2):111–8. doi: 10.1016/S0304-3835(01)00655-3 11566484

[25] Takahashi M Sung B Shen Y Hur K Link A Boland CR. Boswellic Acid Exerts Antitumor Effects in Colorectal Cancer Cells by Modulating Expression of the Let-7 and miR-200 microRNA Family. Carcinogenesis (2012) 33(12):2441–9. doi: 10.1093/carcin/bgs286 PMC3510738 22983985

[26] Shen Y Takahashi M Byun HM Link A Sharma N Balaguer F. Boswellic Acid Induces Epigenetic Alterations by Modulating DNA Methylation in Colorectal Cancer Cells. Cancer Biol Ther (2012) 13(7):542–52. doi: 10.4161/cbt.19604 PMC3364790 22415137

[27] Shimura T Sharma P Sharma GG Banwait JK Goel A. Enhanced Anti-Cancer Activity of Andrographis With Oligomeric Proanthocyanidins Through Activation of Metabolic and Ferroptosis Pathways in Colorectal Cancer. Sci Rep (2021) 11(1):7548. doi: 10.1038/s41598-021-87283-y 33824419 PMC8024269

[28] Zhao Y Wang C Goel A. Andrographis Overcomes 5-Fluorouracil-Associated Chemoresistance Through Inhibition of DKK1 in Colorectal Cancer. Carcinogenesis (2021) 42(6):814–25. doi: 10.1093/carcin/bgab027 PMC8215595 33822896

[29] Sharma P Shimura T Banwait JK Goel A. Andrographis-Mediated Chemosensitization Through Activation of Ferroptosis and Suppression of Beta-Catenin/Wnt-Signaling Pathways in Colorectal Cancer. Carcinogenesis (2020) 41(10):1385–94. doi: 10.1093/carcin/bgaa090 PMC7566354 32835374

[30] Ravindranathan P Pasham D Goel A. Oligomeric Proanthocyanidins (OPCs) From Grape Seed Extract Suppress the Activity of ABC Transporters in Overcoming Chemoresistance in Colorectal Cancer Cells. Carcinogenesis (2019) 40(3):412–21. doi: 10.1093/carcin/bgy184 PMC6514448 30596962

[31] Ravindranathan P Pasham D Balaji U Cardenas J Gu J Toden S. A Combination of Curcumin and Oligomeric Proanthocyanidins Offer Superior Anti-Tumorigenic Properties in Colorectal Cancer. Sci Rep (2018) 8(1):13869. doi: 10.1038/s41598-018-32267-8 30218018 PMC6138725

[32] Toden S Ravindranathan P Gu J Cardenas J Yuchang M Goel A. Oligomeric Proanthocyanidins (OPCs) Target Cancer Stem-Like Cells and Suppress Tumor Organoid Formation in Colorectal Cancer. Sci Rep (2018) 8(1):3335. doi: 10.1038/s41598-018-21478-8 29463813 PMC5820273

[33] Ravindranathan P Pasham D Balaji U Cardenas J Gu J Toden S. Mechanistic Insights Into Anticancer Properties of Oligomeric Proanthocyanidins From Grape Seeds in Colorectal Cancer. Carcinogenesis (2018) 39(6):767–77. doi: 10.1093/carcin/bgy034 PMC5972632 29684110

[34] Habtemariam S. The Quest to Enhance the Efficacy of Berberine for Type-2 Diabetes and Associated Diseases: Physicochemical Modification Approaches. Biomedicines (2020) 8(4):90. doi: 10.3390/biomedicines8040090 PMC7235753 32325761

[35] Kwon S Chan AT. Extracting the Benefits of Berberine for Colorectal Cancer. Lancet Gastroenterol Hepatol (2020) 5(3):231–3. doi: 10.1016/S2468-1253(19)30430-3 31926919

[36] Liu Y Hua W Li Y Xian X Zhao Z Liu C. Berberine Suppresses Colon Cancer Cell Proliferation by Inhibiting the SCAP/SREBP-1 Signaling Pathway-Mediated Lipogenesis. Biochem Pharmacol (2020) 174:113776. doi: 10.1016/j.bcp.2019.113776 31874145

[37] Ruan H Zhan YY Hou J Xu B Chen B Tian Y. Berberine Binds Rxrα to Suppress β-Catenin Signaling in Colon Cancer Cells. Oncogene (2017) 36(50):6906–18. doi: 10.1038/onc.2017.296 PMC5735301 28846104

[38] Zhang H Wang X Wang T Chen K Wang H Jia Q. Enhancement of Berberine Hypoglycemic Activity by Oligomeric Proanthocyanidins. Molecules (2018) 23(12):3318. doi: 10.3390/molecules23123318 PMC6321252 30558158

[39] Guan X Zheng X Vong CT Zhao J Xiao J Wang Y. Combined Effects of Berberine and Evodiamine on Colorectal Cancer Cells and Cardiomyocytes In Vitro. Eur J Pharmacol (2020) 875:173031. doi: 10.1016/j.ejphar.2020.173031 32109457

[40] Wang XN Han X Xu LN Yin LH Xu YW Qi Y. Enhancement of Apoptosis of Human Hepatocellular Carcinoma SMMC-7721 Cells Through Synergy of Berberine and Evodiamine. Phytomedicine (2008) 15(12):1062–8. doi: 10.1016/j.phymed.2008.05.002 18579357

[41] Rather MA Bhat BA Qurishi MA. Multicomponent Phytotherapeutic Approach Gaining Momentum: Is the "One Drug to Fit All" Model Breaking Down? Phytomedicine (2013) 21(1):1–14. doi: 10.1016/j.phymed.2013.07.015 24035674

[42] Zhang A Sun H Wang X. Potentiating Therapeutic Effects by Enhancing Synergism Based on Active Constituents From Traditional Medicine. Phytother Res (2014) 28(4):526–33. doi: 10.1002/ptr.5032 23913598

[43] Dai B Ma Y Yang T Fan M Yu R Su Q. Synergistic Effect of Berberine and HMQ1611 Impairs Cell Proliferation and Migration by Regulating Wnt Signaling Pathway in Hepatocellular Carcinoma. Phytother Res (2019) 33(3):745–55. doi: 10.1002/ptr.6267 30565332

[44] Pinto-Garcia L Efferth T Torres A Hoheisel JD Youns M. Berberine Inhibits Cell Growth and Mediates Caspase-Independent Cell Death in Human Pancreatic Cancer Cells. Planta Med (2010) 76(11):1155–61. doi: 10.1055/s-0030-1249931 20455200

[45] Chou TC. Drug Combination Studies and Their Synergy Quantification Using the Chou-Talalay Method. Cancer Res (2010) 70(2):440–6. doi: 10.1158/0008-5472.CAN-09-1947 20068163

[46] Serafim TL Oliveira PJ Sardao VA Perkins E Parke D Holy J. Different Concentrations of Berberine Result in Distinct Cellular Localization Patterns and Cell Cycle Effects in a Melanoma Cell Line. Cancer Chemother Pharmacol (2008) 61(6):1007–18. doi: 10.1007/s00280-007-0558-9 17661039

[47] Wang S An J Dong W Wang X Sheng J Jia Y. Glucose-Coated Berberine Nanodrug for Glioma Therapy Through Mitochondrial Pathway. Int J Nanomed (2020) 15:7951–65. doi: 10.2147/IJN.S213079 PMC7569050 33116511

[48] Livak KJ Schmittgen TD. Analysis of Relative Gene Expression Data Using Real-Time Quantitative PCR and the 2(-Delta Delta C(T)) Method. Methods (2001) 25(4):402–8. doi: 10.1006/meth.2001.1262 11846609

[49] Harrow J Frankish A Gonzalez JM Tapanari E Diekhans M Kokocinski F. GENCODE: The Reference Human Genome Annotation for The ENCODE Project. Genome Res (2012) 22(9):1760–74. doi: 10.1101/gr.135350.111 PMC3431492 22955987

[50] Kim D Langmead B Salzberg SL. HISAT: A Fast Spliced Aligner With Low Memory Requirements. Nat Methods (2015) 12(4):357–60. doi: 10.1038/nmeth.3317 PMC4655817 25751142

[51] Li H Handsaker B Wysoker A Fennell T Ruan J Homer N. The Sequence Alignment/Map Format and SAMtools. Bioinformatics (2009) 25(16):2078–9. doi: 10.1093/bioinformatics/btp352 PMC2723002 19505943

[52] Anders S Pyl PT Huber W. HTSeq–a Python Framework to Work With High-Throughput Sequencing Data. Bioinformatics (2015) 31(2):166–9. doi: 10.1093/bioinformatics/btu638 PMC4287950 25260700

[53] Love MI Huber W Anders S. Moderated Estimation of Fold Change and Dispersion for RNA-Seq Data With Deseq2. Genome Biol (2014) 15(12):550. doi: 10.1186/s13059-014-0550-8 25516281 PMC4302049

[54] Kanda Y. Investigation of the Freely Available Easy-to-Use Software 'EZR' for Medical Statistics. Bone Marrow Transplant (2013) 48(3):452–8. doi: 10.1038/bmt.2012.244 PMC3590441 23208313

[55] Grassilli E Salomoni P Perrotti D Franceschi C Calabretta B. Resistance to Apoptosis in CTLL-2 Cells Overexpressing B-Myb is Associated With B-Myb-Dependent Bcl-2 Induction. Cancer Res (1999) 59(10):2451–6. 10344757

[56] Selvakumaran M Lin HK Sjin RT Reed JC Liebermann DA Hoffman B. The Novel Primary Response Gene MyD118 and the Proto-Oncogenes Myb, Myc, and Bcl-2 Modulate Transforming Growth Factor Beta 1-Induced Apoptosis of Myeloid Leukemia Cells. Mol Cell Biol (1994) 14(4):2352–60. doi: 10.1128/mcb.14.4.2352-2360.1994 PMC358602 8139540

[57] Biroccio A Benassi B D'Agnano I D'Angelo C Buglioni S Mottolese M. C-Myb and Bcl-X Overexpression Predicts Poor Prognosis in Colorectal Cancer: Clinical and Experimental Findings. Am J Pathol (2001) 158(4):1289–99. doi: 10.1016/S0002-9440(10)64080-1 PMC1891926 11290547

[58] Drabsch Y Robert RG Gonda TJ. MYB Suppresses Differentiation and Apoptosis of Human Breast Cancer Cells. Breast Cancer Res (2010) 12(4):R55. doi: 10.1186/bcr2614 20659323 PMC2949644

[59] Qu X Yan X Kong C Zhu Y Li H Pan D. C-Myb Promotes Growth and Metastasis of Colorectal Cancer Through C-Fos-Induced Epithelial-Mesenchymal Transition. Cancer Sci (2019) 110(10):3183–96. doi: 10.1111/cas.14141 PMC6778643 31338937

[60] Fan X Wang Y Jiang T Liu T Jin Y Du K. B-Myb Accelerates Colorectal Cancer Progression Through Reciprocal Feed-Forward Transactivation of E2F2. Oncogene (2021) 40(37):5613–25. doi: 10.1038/s41388-021-01961-9 PMC8445821 34316028

[61] Cicirò Y Sala A. MYB Oncoproteins: Emerging Players and Potential Therapeutic Targets in Human Cancer. Oncogenesis (2021) 10(2):19. doi: 10.1038/s41389-021-00309-y 33637673 PMC7910556

[62] Mitra P. Transcription Regulation of MYB: A Potential and Novel Therapeutic Target in Cancer. Ann Transl Med (2018) 6(22):443. doi: 10.21037/atm.2018.09.62 30596073 PMC6281535

[63] Weeber F Ooft SN Dijkstra KK Voest EE. Tumor Organoids as a Pre-Clinical Cancer Model for Drug Discovery. Cell Chem Biol (2017) 24(9):1092–100. doi: 10.1016/j.chembiol.2017.06.012 28757181

[64] Newman DJ Cragg GM. Natural Products as Sources of New Drugs Over the Nearly Four Decades From 01/1981 to 09/2019. J Nat Prod (2020) 83(3):770–803. doi: 10.1021/acs.jnatprod.9b01285 32162523

[65] Xu Z Du P Meiser P Jacob C. Proanthocyanidins: Oligomeric Structures With Unique Biochemical Properties and Great Therapeutic Promise. Nat Prod Commun (2012) 7(3):381–8. doi: 10.1177/1934578X1200700321 22545414

[66] Tariq A Sadia S Pan K Ullah I Mussarat S Sun F. A Systematic Review on Ethnomedicines of Anti-Cancer Plants. Phytother Res (2017) 31(2):202–64. doi: 10.1002/ptr.5751 28093828

[67] Zhao BX Sun YB Wang SQ Duan L Huo QL Ren F. Grape Seed Procyanidin Reversal of P-Glycoprotein Associated Multi-Drug Resistance via Down-Regulation of NF-κb and MAPK/ERK Mediated YB-1 Activity in A2780/T Cells. PloS One (2013) 8(8):e71071. doi: 10.1371/journal.pone.0071071 23967153 PMC3744527

[68] Huang J Huang J Feng W Li S Tang H Qin S Li W. Berberine Exerts Anti-Cancer Activity by Modulating Adenosine Monophosphate- Activated Protein Kinase (AMPK) and the Phosphatidylinositol 3-Kinase/ Protein Kinase B (PI3K/AKT) Signaling Pathways. Curr Pharm Des (2021) 27(4):565–74. doi: 10.2174/1381612826666200928155728 32988344

[69] Farooqi AA Qureshi MZ Khalid S Attar R Martinelli C Sabitaliyevich UY. Regulation of Cell Signaling Pathways by Berberine in Different Cancers: Searching for Missing Pieces of an Incomplete Jig-Saw Puzzle for an Effective Cancer Therapy. Cancers (Basel) (2019) 11(4):478. doi: 10.3390/cancers11040478 PMC6521278 30987378

[70] Chen ZZ. Berberine Induced Apoptosis of Human Osteosarcoma Cells by Inhibiting Phosphoinositide 3 Kinase/Protein Kinase B (PI3K/Akt) Signal Pathway Activation. Iran J Public Health (2016) 45(5):578–85. PMC4935701 27398330

[71] Kuo HP Chuang TC Yeh MH Hsu SC Way TD Chen PY. Growth Suppression of HER2-Overexpressing Breast Cancer Cells by Berberine via Modulation of the HER2/PI3K/Akt Signaling Pathway. J Agric Food Chem (2011) 59(15):8216–24. doi: 10.1021/jf2012584 21699261

[72] Li G Zhang C Liang W Zhang Y Shen Y Tian X. Berberine Regulates the Notch1/PTEN/PI3K/AKT/mTOR Pathway and Acts Synergistically With 17-AAG and SAHA in SW480 Colon Cancer Cells. Pharm Biol (2021) 59(1):21–30. doi: 10.1080/13880209.2020.1865407 33417512 PMC7808376

