# Corrigendum: Berberine and Oligomeric Proanthocyanidins Exhibit Synergistic Efficacy Through Regulation of PI3K-Akt Signaling Pathway in Colorectal Cancer

**DOI:** 10.3389/fonc.2022.952180

**Published:** 2022-06-29

**Authors:** Keisuke Okuno, Rachana Garg, Yate-Ching Yuan, Masanori Tokunaga, Yusuke Kinugasa, Ajay Goel

**Affiliations:** ^1^ Department of Molecular Diagnostics and Experimental Therapeutics, Beckman Research Institute of City of Hope, Biomedical Research Center, Monrovia, CA, United States; ^2^ Department of Gastrointestinal Surgery, Tokyo Medical and Dental University, Tokyo, Japan; ^3^ Translational Bioinformatics, Center for Informatics, City of Hope, Duarte, CA, United States; ^4^ City of Hope Comprehensive Cancer Center, Duarte, CA, United States

**Keywords:** colorectal cancer, berberine, oligomeric proanthocyanidins, grape seed extract, synergistic effect, apoptosis, MYB, PI3K-Akt signaling pathway

In the published article, there was an error about the source of berberine. A correction has been made to the section of **Materials and Methods**, “*Cell Culture and Materials”*, paragraph 1:

**“**Grape seed-OPCs (VX1 extract, EuroPharma USA, Green Bay, WI) and berberine (Berberine HCL, EuroPharma USA, Green Bay, WI) were dissolved in DMSO**”**.

The authors apologize for this error and state that this does not change the scientific conclusions of the article in any way. The original article has been updated.

## Publisher’s Note

All claims expressed in this article are solely those of the authors and do not necessarily represent those of their affiliated organizations, or those of the publisher, the editors and the reviewers. Any product that may be evaluated in this article, or claim that may be made by its manufacturer, is not guaranteed or endorsed by the publisher.

