# Supplementary figures and images for: Berberine and Oligomeric Proanthocyanidins Exhibit Synergistic Efficacy Through Regulation of PI3K-Akt Signaling Pathway in Colorectal Cancer
Source: Front Oncol. 2022 May 4;12:855860. doi: 10.3389/fonc.2022.855860 (PMC9114748; doi:10.3389/fonc.2022.855860)

# Supplementary Figure S1

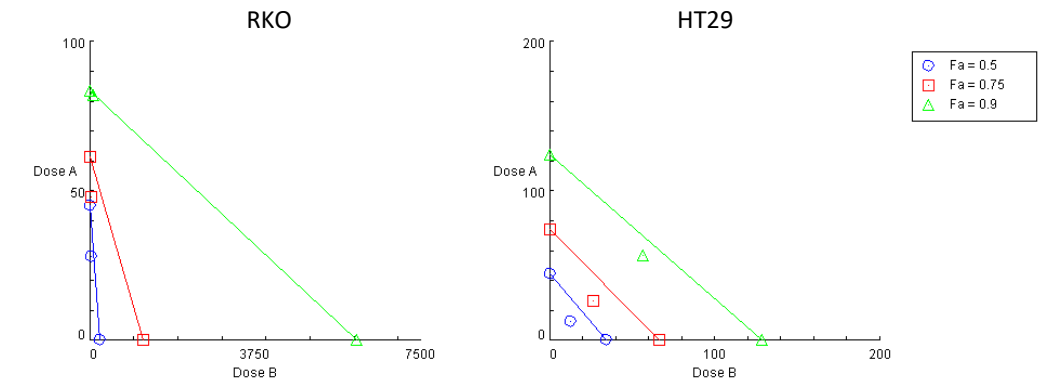

# Supplementary Figure S2

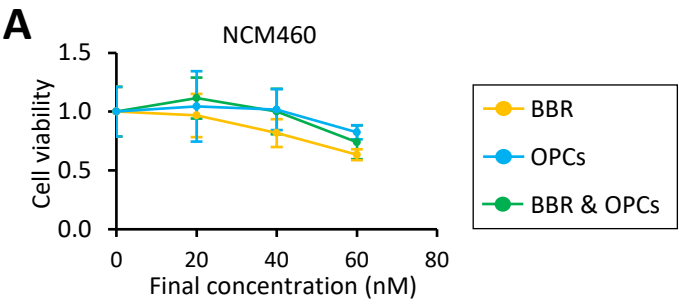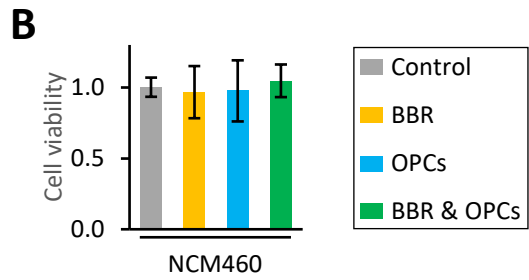

Supplementary Figure S3

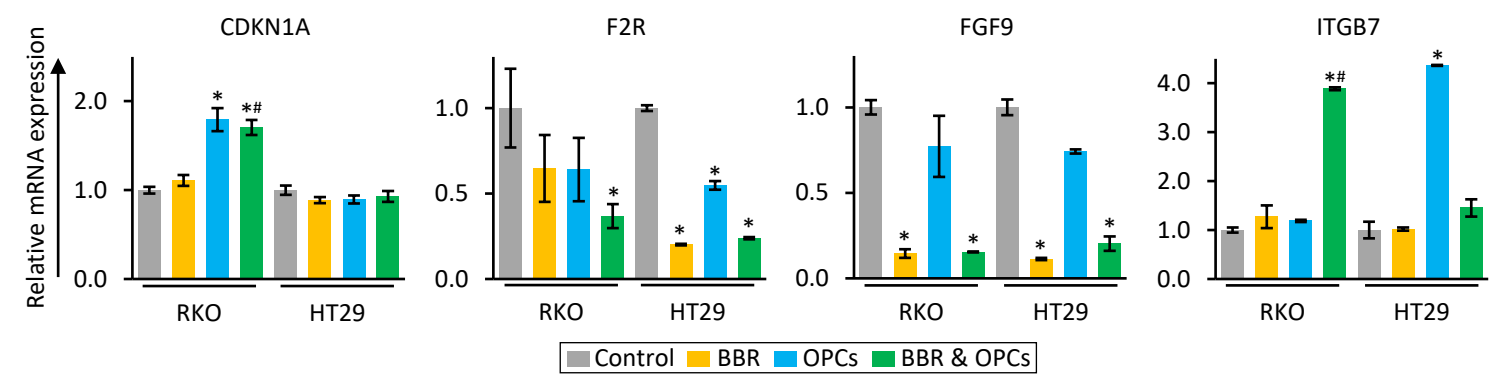

Supplement: Supplementary Figure 1 — Isobologram analysis after combined treatment with BBR and OPCs in RKO and HT29 cells. [file DataSheet_1.pdf]
